# Integrative psychological interventions for stress regulation in sport: a mini-review

**DOI:** 10.3389/fspor.2026.1798062

**Published:** 2026-04-23

**Authors:** Walaa Jumah Alkasasbeh, Gerasimos V. Grivas, Adam Tawfiq Amawi

**Affiliations:** 1 Department of Physical Education, School of Sports Science, The University of Jordan, Amman, Jordan; 2 Physical Education and Sports, Division of Humanities and Political Sciences, Hellenic Naval Academy, Piraeus, Greece; 3 Department of Movement Sciences and Sports Training, School of Sports Science, The University of Jordan, Amman, Jordan

**Keywords:** athlete well-Being, cognitive-behavioral interventions, digital health in sport, Hrv biofeedback, mindfulness, sport psychology, stress regulation, sustainable athlete development

## Abstract

Psychological stress plays a critical role in shaping performance and well-being in competitive sport. This mini-review examines three major categories of psychological interventions for stress regulation in athletes: cognitive-behavioral approaches, mindfulness- and acceptance-based interventions, and psychophysiological and technology-assisted strategies. While existing literature generally supports the effectiveness of these approaches in improving emotional regulation, attentional control, and stress management, the evidence remains heterogeneous and often context-dependent. Importantly, current findings suggest that no single intervention can be considered universally optimal, as effectiveness appears to vary according to athlete characteristics and sport-specific demands. This review provides a critical and comparative synthesis of the literature, highlighting differential roles of intervention approaches across performance contexts. Cognitive-behavioral strategies appear most effective for structured pre-competition preparation, mindfulness-based approaches for in-performance regulation, and psychophysiological and technology-assisted strategies for monitoring and recovery processes. In addition, the increasing integration of technology in sport presents both opportunities and challenges. While technology-assisted tools may enhance self-awareness and feedback, excessive reliance on external monitoring may limit the development of autonomous self-regulation skills, particularly among younger athletes. Overall, stress regulation is best conceptualized as a trainable, context-sensitive skill that requires individualized and integrative intervention approaches. Future research should focus on comparative and longitudinal designs to better understand the interaction between intervention type, athlete profile, and performance context.

## 1 Introduction

Psychological stress is an inherent component of competitive sport and represents a critical factor influencing athletic performance, mental well-being, and long-term engagement in sport (1). *Psychophysiological stress responses in elite athletes fluctuate across competitions and relate to performance, highlighting the link between stress regulation and competitive demands* (2). Athletes are frequently exposed to multiple stressors, including performance expectations (3), training load (4), competitive pressure (5), injury risk (6), and organizational demands (7). When inadequately regulated, psychological stress can impair concentration (8), decision-making (9, 10), emotional control (11), and physiological functioning (12), ultimately compromising performance outcomes.

From a theoretical perspective, this review is grounded in the transactional model of stress proposed by Richard Lazarusand Susan Folkman, which conceptualizes stress as a dynamic process emerging from the interaction between environmental demands and individual cognitive appraisal processes (13). Within this framework, stress responses are determined not by the objective characteristics of the situation, but by how athletes evaluate perceived demands in relation to their coping resources (14). This model provides a conceptual foundation for understanding why psychological interventions targeting appraisal, coping, and self-regulation processes are central to effective stress regulation in sport (15).

Early research in sport psychology largely focused on identifying sources of stress and describing athletes’ stress responses (16–18). However, recent advances have shifted attention toward the development and application of psychological interventions aimed at regulating stress rather than merely documenting its presence (19). This shift reflects a growing recognition that effective stress regulation is not only essential for optimizing performance but also for supporting athlete health and sustainable participation in sport (19, 20).

Contemporary psychological interventions increasingly adopt integrative frameworks, reflecting a conceptual shift away from reductionist, single-component models toward multidimensional approaches to stress regulation (21). Such integrative interventions simultaneously engage cognitive appraisal processes, emotional regulation mechanisms, and physiological activation systems, thereby offering a more ecologically valid representation of how athletes experience and regulate stress in competitive environments. Within this perspective, cognitive-behavioral strategies may primarily support pre-competition cognitive preparation (22), mindfulness- and acceptance-based techniques facilitate in-performance attentional and emotional regulation (23), and psychophysiological strategies, including biofeedback and digital technologies, contribute to post-performance recovery and self-regulation processes (24).

Despite the growing body of literature supporting the effectiveness of psychological interventions for stress regulation in sport (19, 25–27), the current evidence remains heterogeneous in terms of study design, intervention characteristics, and outcome measures. Moreover, the effectiveness of these approaches does not appear to be uniform across different sport contexts, as it may vary depending on factors such as athlete characteristics, competitive level, and the specific nature of performance demands. In addition, the increasing integration of technology into sport environments introduces further complexity (28, 29), raising questions about the role of contemporary tools in comparison to traditional psychological methods.

Accordingly, this mini-review provides a critical and comparative perspective, highlighting the context-dependent effectiveness of different intervention approaches and their differential roles across athlete profiles and performance contexts, thereby contributing to a more nuanced understanding of stress regulation in sport.

## 2 Methodology

This study adopted a narrative mini-review design to analyze and organize the literature related to psychological stress regulation in competitive sport contexts, with an emphasis on integrating theoretical frameworks, correlational studies, applied research, and psychological and psychophysiological interventions relevant to the topic. This approach aims to provide a structured analytical overview that reflects the diversity of approaches used to understand sport-related stress and its regulatory mechanisms.

A targeted search was conducted across major academic databases, including PubMed, Scopus, and Google Scholar, using combinations of the following keywords: psychological stress, stress regulation, sport psychology, cognitive-behavioral interventions, mindfulness, acceptance-based interventions, heart rate variability (HRV) biofeedback, and technology-assisted interventions. Reference lists of relevant studies were also screened to identify additional sources pertinent to the topic.

Studies were selected based on their relevance to psychological stress or performance-related psychological factors in sport, whether they examined direct psychological or psychophysiological interventions, discussed cognitive and emotional regulatory mechanisms associated with stress responses, or reported outcomes related to sport performance, emotional regulation, cognitive appraisal, burnout, or physiological indicators linked to stress in sport settings. Conceptual and theoretical reviews were also included when necessary to clarify explanatory models of stress and adaptation in sport, without being treated as direct empirical evidence of intervention effectiveness.

The included literature was treated as a continuum of evidence encompassing intervention studies, physiological monitoring and measurement research, correlational investigations, and theoretical contributions, with careful consideration of each study’s design in interpreting findings. Although the primary focus was on athlete populations, selected conceptual, monitoring, and technology-oriented studies from related performance contexts were included when they provided theoretical, methodological, or contextual clarification relevant to stress regulation in sport. The studies were thematically organized according to major domains reflecting prevailing directions in the field, including cognitive-behavioral approaches, mindfulness- and acceptance-based interventions, and psychophysiological and technology-assisted approaches, in order to provide a coherent analytical synthesis aligned with the nature of the included literature.

## 3 Stress in sport: brief conceptual overview

Psychological stress in sport is commonly understood as a dynamic process resulting from the interaction between performance demands and the psychological resources available to the athlete (30, 31). In this review, psychological resources are defined as cognitive-regulatory capacities, emotional regulation abilities, and adaptive coping skills that support athletes in managing performance demands (32–34). Stress does not arise solely from the competitive situation itself, but from how athletes perceive and evaluate these demands in relation to their coping abilities (35), making stress a highly individualized experience.

Importantly, stress regulation in sport should be understood as a context-dependent and individualized process rather than a uniform response across athletes. Variations in athlete characteristics such as age, competitive level, gender, and cultural background may significantly influence both stress appraisal and the effectiveness of regulatory strategies (36–39). For instance, younger or less experienced athletes may rely more on structured guidance and externally supported coping techniques (40), whereas elite athletes may benefit from more flexible and self-directed regulation strategies (41). Similarly, gender-related differences in emotional processing and sociocultural expectations may shape preferred coping styles and responses to stress (42). Cultural context may further influence how stress is perceived, expressed, and managed, as well as the acceptability of certain psychological interventions (43). In addition, the nature of sport-specific demands, including the intensity of physical exertion and cognitive load, may influence athletes' psychophysiological responses and potentially determine the most effective regulation strategies (44). For example, high-arousal, physically demanding sports may require different regulatory approaches compared to precision-based sports that emphasize attentional control (45). In precision sports such as shooting, performance is highly dependent on effective emotional regulation and stability under pressure (46).

In addition, stress in competitive sport is not uniform, but may stem from diverse sources such as competitive pressure (47), organizational demands (48), injury-related uncertainty (49), and chronic training load stress (50). Additionally, interpersonal adversities such as psychological abuse and neglect have been identified among national-level athletes, representing chronic relational stressors that may further compound performance-related pressure and affect well-being (51). In this context, it is also important to distinguish between coping and stress regulation (18), which are sometimes used interchangeably but represent different theoretical processes. Coping generally refers to the cognitive and behavioral efforts athletes use to manage external demands or stressors (52), often involving behavioral or strategic responses such as problem-solving, self-talk, or seeking support. While some theoretical models view emotion regulation as part of coping (53), this review adopts a functional distinction for clarity. Coping is conceptualized as situation-oriented efforts to manage external stressors (18), whereas stress regulation refers more broadly to the modulation of internal cognitive, emotional, and physiological processes underlying the stress response (21). Evidence from elite basketball referees further indicates that emotional regulation capacity is positively associated with officiating performance under competitive pressure, supporting the functional relevance of internal regulatory mechanisms (54). This differentiation facilitates clearer identification of interventions targeting coping strategies versus those addressing underlying regulatory mechanisms.

Such conceptual clarification is particularly important for later comparisons between CBT-based approaches, which frequently strengthen coping strategies, and mindfulness-based approaches, which primarily emphasize internal regulation and acceptance under pressure. Stress regulation refers more specifically to the modification of internal psychological and physiological processes (55), such as emotional balance (56), attentional control (57), and autonomic activation in response to stress (58). Evidence from handball players further suggests that prevailing mood patterns such as psychological flexibility and excitement-related states may reflect pre-competition affective readiness, reinforcing the role of emotional regulation processes within performance contexts (59).‏ Within the transactional framework, stress is an appraisal-based process (60), whereas anxiety is a potential emotional response to that process (61). These distinctions are critical, as intervention effectiveness may vary depending on the dominant stressor type and the competitive context in which it occurs.

Contemporary conceptualizations emphasize the role of cognitive appraisal in shaping stress responses (62). Athletes may interpret competitive situations as challenges or threats, which in turn influences emotional, physiological, and behavioral responses such as anxiety, arousal regulation, and effort allocation during performance (1, 63, 64).

Within this framework, stress regulation is viewed as a learnable psychological skill that can be enhanced through targeted psychological interventions (21). By improving appraisal processes and strengthening psychological resources, athletes can better adapt to training and competition demands (35). This conceptual understanding provides the foundation for integrative psychological interventions aimed at optimizing performance and supporting athlete well-being.

## 4 Recent psychological interventions

### 4.1 Cognitive-behavioral approaches

Cognitive-behavioral approaches represent one of the most widely used psychological interventions for stress regulation in sport (65). These interventions are grounded in the premise that stress responses are strongly influenced by athletes' thoughts, beliefs, and interpretations of performance-related situations (66). By targeting maladaptive cognitive patterns, cognitive-behavioral interventions aim to enhance athletes' ability to cope with competitive demands (67).

Common techniques include cognitive restructuring (68), which helps athletes identify and modify irrational or negative performance-related thoughts (69), and coping skills training, including structured self-talk interventions (instructional and cognitive restructuring–based), goal setting, and imagery (70). Within this framework, CBT-based approaches may also contribute to the enhancement of performance-related confidence and self-efficacy (71), as modifying maladaptive cognitions and reinforcing mastery experiences can strengthen athletes' perceived competence under pressure (72, 73). In addition, several CBT-informed protocols incorporate elements of arousal regulation (74), including relaxation and controlled breathing techniques, particularly in contexts characterized by hyperactivation or over-arousal (75). Performance routines, when grounded in cognitive restructuring and attentional control principles, are typically integrated within CBT-based psychological skills training rather than conceptualized as entirely distinct interventions.

These strategies have been associated with reductions in performance-related anxiety (76), improve emotional control (77), and promote more adaptive responses to stress during training and competition (35). However, effect sizes appear to vary across sport type, competitive level, and intervention duration. Additionally, many studies rely primarily on self-reported psychological outcomes, and fewer investigations directly assess objective performance indicators or long-term maintenance effects. Overall, cognitive-behavioral approaches provide structured and practical tools that can be integrated into sport training programs. Nevertheless, their effectiveness is likely influenced by contextual moderators such as athlete readiness, practitioner expertise, and the specific demands of the competitive environment, suggesting the need for continued applied and ecologically valid research.

However, the effectiveness of cognitive-behavioral approaches may not be uniform across all sport contexts and athlete populations (78). Psychological factors such as coping strategies and resilience play a key role in managing pre-competition anxiety, highlighting the importance of structured psychological preparation prior to competition (79). In contrast, their applicability may be more limited in highly dynamic and unpredictable performance environments that require rapid, flexible responses under pressure (80). Furthermore, the effectiveness of CBT-based approaches often depends on the athlete’s level of cognitive awareness (81), motivation (82), and willingness to engage in reflective processes (83), which may vary across developmental stages and competitive levels. In addition, a notable proportion of the existing literature relies on self-reported psychological outcomes, such as perceived anxiety or emotional control (84), with fewer studies examining objective performance indicators or long-term transfer effects. This raises important questions regarding the extent to which observed psychological improvements translate into consistent performance enhancement.

Therefore, while cognitive-behavioral approaches remain a cornerstone of psychological skills training in sport, their effectiveness should be considered context-dependent rather than universally superior across all situations. Compared to mindfulness-based approaches, cognitive-behavioral strategies tend to be more structured and may be particularly effective during pre-competition preparation.

### 4.2 Mindfulness and acceptance-based interventions

Mindfulness- and acceptance-based interventions are increasingly used in sport psychology to support stress regulation in competitive contexts (85). These approaches focus on enhancing athletes' awareness of internal experiences (86), such as thoughts, emotions, and physiological sensations (87), without attempting to suppress or control them.

Mindfulness-based practices encourage present-moment attention and nonjudgmental observation (88), allowing athletes to respond to stress with greater emotional balance and attentional stability. Such interventions have been associated with reductions in rumination and improvements in emotional regulation during high-pressure performance situations (89). However, the magnitude of these effects appears to depend on intervention duration, athlete engagement, and baseline psychological characteristics. Several studies are conducted with relatively small samples or short intervention periods, which may limit generalizability across sport contexts.

Acceptance-based approaches, including Acceptance and Commitment Therapy (ACT), emphasize psychological flexibility by helping athletes accept stress-related discomfort while maintaining focus on performance-relevant behaviors (90). By promoting adaptive responses to inevitable competitive stressors rather than attempts to control internal experiences, mindfulness and acceptance-based interventions may offer practical and transferable skills that can be integrated into training and competition settings (91).

Overall, mindfulness- and acceptance-based interventions appear to enhance aspects of stress regulation, particularly emotional balance and psychological flexibility; however, improvements in psychological processes do not always translate directly into consistent objective performance gains, and further ecologically valid research is warranted.

However, the effectiveness of mindfulness- and acceptance-based interventions may vary depending on the specific performance context and athlete characteristics. These approaches may be particularly beneficial in situations that require sustained attentional focus and present-moment awareness under pressure, such as during in-competition performance. At the same time, their impact on objective performance outcomes remains less consistent, as improvements in psychological flexibility and emotional regulation do not always directly translate into measurable performance enhancement. Furthermore, the effectiveness of these interventions is often influenced by factors such as athlete engagement, adherence to practice, and baseline psychological readiness (92). Many mindfulness-based programs require regular and sustained practice, which may present challenges for athletes with limited time or lower motivation (93).‏

These considerations suggest that mindfulness- and acceptance-based interventions may be more suitable for certain athlete profiles and performance demands rather than being universally optimal, highlighting the importance of context-sensitive implementation in applied sport settings. In contrast to cognitive-behavioral approaches, mindfulness-based interventions may be particularly suited to in-performance regulation contexts that require attentional flexibility and present-moment awareness.

### 4.3 Psychophysiological and technology-assisted interventions

Psychophysiological and technology-assisted interventions have emerged as practical tools for regulating stress in sport by targeting the interaction between psychological processes and physiological responses (94). These approaches aim to enhance athletes' awareness and control of stress-related physiological activation, potentially supporting more adaptive performance under pressure (95).

One commonly discussed psychophysiological approach in sport and performance contexts is heart rate variability (HRV). Structured HRV biofeedback protocols involve resonance-frequency breathing designed to stimulate respiratory sinus arrhythmia and baroreflex mechanisms (96). However, beyond biofeedback applications, HRV is more frequently used as a non-invasive monitoring tool to assess autonomic function, training load, recovery status, and physiological readiness in athletic and operational populations (97, 98). In parallel, broader psychophysiological research has examined multiple biosignals including ECG, EDA, EMG, EEG, and respiratory indices as objective markers of stress detection and autonomic activation, primarily for assessment and monitoring purposes rather than as direct intervention strategies (99).

Although HRV biofeedback represents one of the most discussed psychophysiological modalities, it is important to differentiate between HRV as a biomarker of emotional regulation processes (100) and its use as an active training intervention. In some athletic studies, HRV has been employed primarily as a physiological index rather than as a structured biofeedback protocol (101). Technology-supported tools, such as mobile applications in aquatic settings, have shown potential in reducing psychological barriers such as fear (102). More direct evidence for intervention effects stems from randomized trials employing multimodal biofeedback protocols, which have reported preliminary improvements in autonomic functioning, sustained attention, cognitive performance, and reductions in state anxiety among competitive athletes (103, 104). Nevertheless, these trials remain limited in scale and duration.

Overall, psychophysiological approaches in sport range from physiological monitoring to structured biofeedback interventions. While monitoring tools provide valuable insights into readiness and recovery, experimental evidence for direct performance enhancement remains limited and context-dependent, highlighting the need for more rigorous applied research.

In addition to traditional biofeedback approaches, digital technologies have increasingly been used to support physiological monitoring and training processes in sport contexts. Several studies and reviews highlight the role of wearable devices in continuously assessing biomechanical and physiological parameters, including heart rate and workload indicators, primarily for monitoring and training management purposes rather than direct psychological intervention (105–107). Similarly, broader lifestyle and health-related investigations have examined associations between behavioral factors and psychophysiological variables without implementing structured stress-regulation interventions (108, 109).

Mobile-based applications and digital platforms have also been explored as potential tools for delivering psychological skills content; however, current evidence indicates substantial variability in app quality and limited empirical validation of their effectiveness (110). Experimental evidence for technology-assisted interventions remains emerging. For example, technology-aided training has demonstrated improvements in certain physical performance indicators, although psychological outcomes appear dependent on individual factors such as sport involvement (94). Virtual reality environments have been used to simulate competitive pressure in ecologically valid experimental designs, enabling the examination of anxiety and performance responses under controlled stress exposure (111). Overall, while digital and wearable technologies provide scalable tools for monitoring physiological and performance-related variables, robust evidence supporting consistent psychological stress-regulation benefits remains limited and context-dependent.

Importantly, it is necessary to distinguish between different categories of psychophysiological and technology-assisted approaches, as they do not serve identical functions. Physiological monitoring tools primarily provide objective feedback on autonomic and performance-related states (112), whereas biofeedback interventions aim to actively train regulatory control over physiological processes (113). In contrast, digital delivery platforms are often used to disseminate psychological skills content (114), while immersive technologies such as virtual reality are designed to simulate performance environments and stress exposure (115). This distinction is critical, as the mechanisms and expected outcomes of these approaches differ substantially. Although these technologies are increasingly promoted as innovative solutions for stress regulation, the current evidence base remains uneven. While they offer scalability, real-time feedback, and potential for personalized interventions, empirical support for their consistent effectiveness in improving psychological stress regulation and performance outcomes is still developing. In many cases, observed benefits are context-specific, and findings are derived from small-scale or short-term studies, limiting generalizability.

Furthermore, the growing reliance on technology in sport raises important conceptual questions regarding its role in psychological skill development. On one hand, external feedback provided by technological tools may enhance self-awareness and facilitate the learning of regulation strategies (116), particularly in early stages of skill acquisition. On the other hand, excessive reliance on continuous monitoring and external data may reduce athletes' ability to internally regulate stress without technological support (117). This potential tension between technological assistance and psychological autonomy suggests that such tools should be integrated as complementary supports rather than replacements for foundational psychological skills training.

Taken together, contemporary psychological approaches to stress regulation in sport reflect a multidimensional perspective that addresses cognitive, emotional, and physiological processes underlying performance under pressure. Cognitive-behavioral strategies primarily target appraisal and coping mechanisms, mindfulness- and acceptance-based approaches emphasize psychological flexibility and emotional awareness, while psychophysiological and technology-assisted methods contribute to autonomic monitoring and, in some cases, structured regulation training. Compared to traditional psychological approaches, technology-assisted strategies primarily support monitoring and feedback processes rather than directly enhancing core self-regulation skills. Although these approaches appear conceptually complementary, empirical validation of fully integrated intervention models remains limited, and direct comparative trials are still scarce.

**Figure 1:**
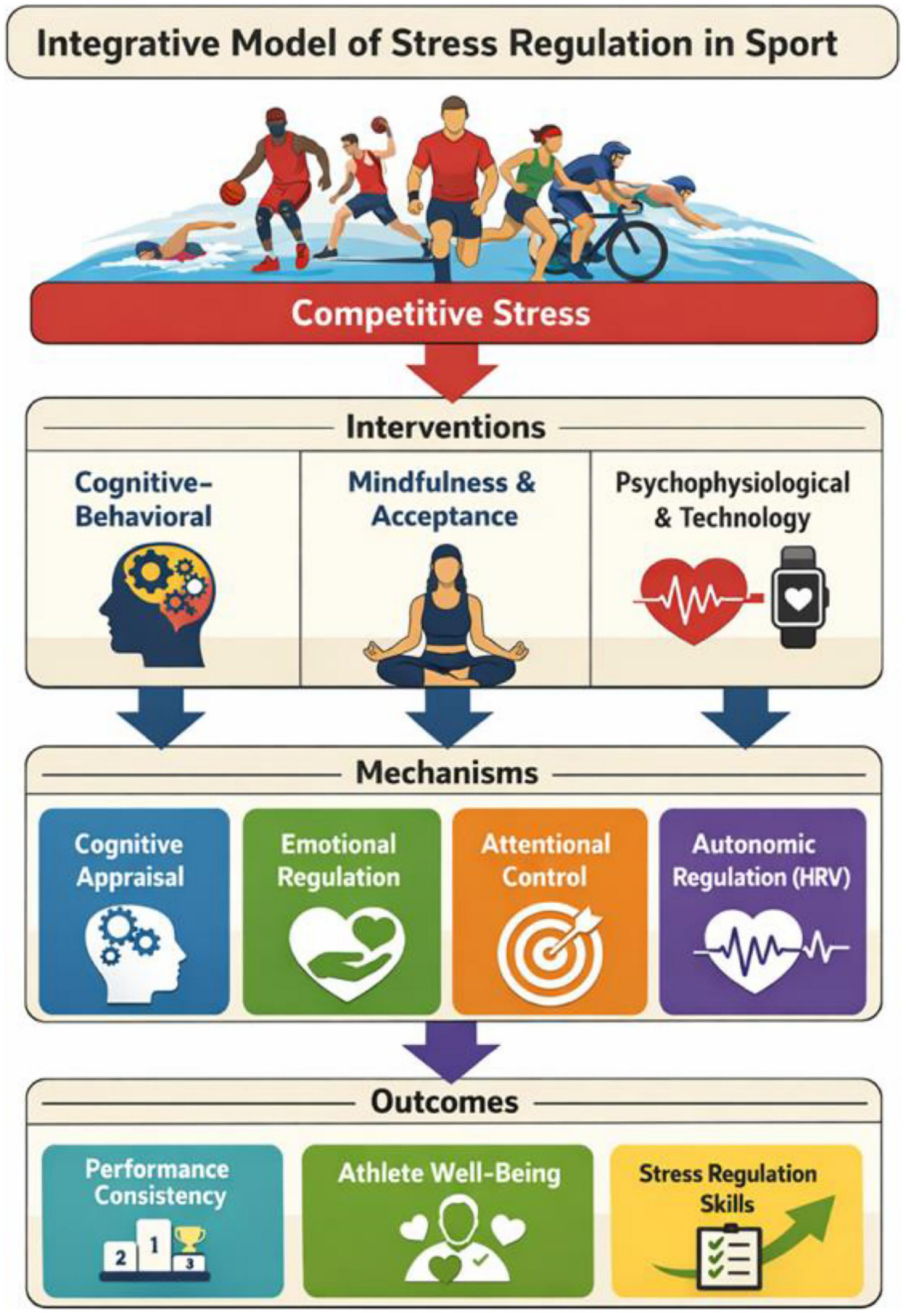
Integrative Model of Psychological Stress Regulation in Sport.

Figure 1 presents a conceptual integrative model synthesizing existing literature on psychological stress regulation in sport. The model is not intended to represent empirically validated causal pathways, but rather to illustrate theoretically proposed relationships. Stress regulation processes are inherently dynamic and reciprocal, and feedback interactions between mechanisms and outcomes may occur in applied sport contexts.

## 5 Practical implications

The findings highlighted in this mini-review emphasize the importance of integrating psychological stress regulation strategies into daily training and competitive preparation rather than treating them as isolated interventions. Cognitive-behavioral, mindfulness-based, and psychophysiological approaches can be systematically embedded within training routines to support athletes' ability to manage stress, maintain attentional control, and sustain performance under pressure (81).

In applied sport settings, cognitive-behavioral strategies, including structured self-talk, cognitive reframing, and pre-performance routines, can be incorporated into pre-competition preparation to support adaptive appraisal and coping (79, 118, 119). Mindfulness-based techniques, such as brief breathing practices and present-moment attentional exercises, may be integrated into warm-up protocols or employed during competition to promote emotional balance and attentional stability (120, 121). In parallel, psychophysiological approaches, including HRV biofeedback and wearable monitoring technologies, may be particularly valuable during post-training or recovery periods to facilitate autonomic regulation and enhance athletes' awareness of stress-related physiological activation (122, 123).

Coaches and sport psychologists play a central role in selecting and implementing appropriate interventions based on athletes' individual needs, sport demands, and developmental stage (124, 125). Moreover, motivational climates that foster intrinsic motivation, personal goal orientation, and self-determination may further strengthen athletes' resilience to competitive stress and support sustained performance engagement (126).‏ Implementation may be influenced by sport-specific logistical constraints and resource considerations, including limited training time, competition demands, access to technology-assisted tools, practitioner expertise, and athlete engagement.

The use of brief, skill-based techniques and technology-assisted tools allows for flexible application across training, competition, and recovery phases (127). Adopting an integrative approach to psychological stress regulation not only enhances performance consistency but also supports athlete well-being and long-term engagement in sport (30). While practical integration of stress regulation strategies into daily training routines is promising, implementation may be influenced by contextual constraints such as time availability, organizational culture, resource allocation, and athlete receptivity.

Importantly, the selection and implementation of stress regulation strategies should be tailored to the athlete’s individual profile rather than applied in a standardized manner. Not all athletes benefit equally from the same intervention approaches, as effectiveness may vary depending on factors such as age, competitive level, psychological readiness, and sport-specific demands. For example, less experienced or younger athletes may require more structured and guided interventions, whereas elite athletes may benefit from flexible, self-directed regulation strategies that can be adapted in real-time performance contexts. In addition, the integration of technology-assisted tools should be approached thoughtfully. While wearable devices and digital platforms can enhance monitoring and provide valuable feedback, they should not replace the development of core psychological regulation skills. Coaches and practitioners should aim to use technology as a supportive tool that complements, rather than substitutes, internal self-regulation processes. Particularly in youth sport, the use of technology should be carefully scaffolded to promote awareness and learning, without fostering excessive dependence on external feedback. Ultimately, the goal of psychological stress regulation in sport should extend beyond immediate performance outcomes to include the development of sustainable self-regulation skills that support long-term performance, well-being, and athlete autonomy.

## 6 Future directions

Future research on psychological stress regulation in sport should increasingly focus on the development of personalized and context-specific interventions that account for individual differences in sport type, competitive level, developmental stage, and psychological profiles. Tailoring interventions to athletes' unique stressors and coping resources may enhance both effectiveness and adherence.

In this context, future studies should also incorporate subgroup analyses that explicitly examine how intervention effectiveness varies across factors such as age, gender, competitive level, and sport-specific demands, in order to better understand differential responses to stress regulation strategies.

In addition, greater integration of technology with established psychological approaches represents a promising direction. Combining psychophysiological monitoring tools, such as HRV and wearable devices, with cognitive-behavioral and mindfulness-based strategies may allow for real-time feedback and adaptive intervention delivery within training and competition environments.

However, further research is needed to examine the long-term implications of technology-assisted interventions, particularly in relation to athletes' psychological autonomy and potential dependence on external feedback systems. Longitudinal designs may be especially valuable in clarifying whether these tools support or hinder the development of sustainable self-regulation skills over time.

Finally, there is a need for more applied, short-term, and ecologically valid studies conducted in real-world sport settings. Such research should prioritize practical outcomes, including performance consistency, stress regulation skills, and athlete well-being, to further bridge the gap between theory, research, and applied sport psychology practice.

Future research would also benefit from direct comparative trials examining the relative effectiveness of different intervention approaches such as cognitive-behavioral, mindfulness-based, and psychophysiological methods, under similar conditions. In addition, the use of mixed-method designs may provide deeper insights into not only intervention outcomes but also athletes' subjective experiences, perceptions, and engagement with different stress regulation strategies.

## 7 Critical appraisal of the evidence base

Despite the growing body of literature examining psychological stress-regulation interventions in sport, several methodological considerations warrant careful interpretation of the existing evidence base. Although randomized controlled trials (RCTs) have been conducted in selected domains, particularly within cognitive-behavioral and biofeedback-based interventions, a substantial proportion of studies rely on quasi-experimental, pilot, or single-group designs, which may limit causal inference.

Sample sizes across intervention studies are frequently modest, particularly in elite or sport-specific populations, where recruitment constraints are common. While such samples reflect ecological realities in applied sport contexts, they may reduce statistical power and generalizability across competitive levels and sport types. In addition, intervention duration and follow-up periods vary considerably, with many studies emphasizing short-term psychological outcomes rather than long-term maintenance of stress-regulation skills.

Outcome measurement approaches also demonstrate heterogeneity. A significant number of studies rely on self-reported psychological indicators such as perceived stress, anxiety, and emotional regulation, whereas fewer investigations integrate objective performance metrics or multimodal psychophysiological indices. Although subjective measures are theoretically aligned with appraisal-based stress models, reliance on single-method assessment may increase susceptibility to response bias and shared-method variance.

Importantly, the heterogeneity of study designs, intervention protocols, and outcome measures makes direct comparison across intervention approaches particularly challenging. This variability limits the ability to draw clear conclusions regarding the relative effectiveness of different stress-regulation strategies. In addition, only a limited number of studies explicitly examine how intervention effects may vary across athlete subgroups, such as differences related to age, gender, competitive level, or sport type, which constrains the understanding of context-specific effectiveness.

Furthermore, variability in intervention protocols, including differences in dosage, practitioner expertise, delivery format (individual vs. group), and integration within training routines, complicates direct comparison across studies. This limitation is particularly evident in emerging domains such as technology-assisted and wearable-based interventions, where empirical evidence remains uneven and is often derived from small-scale, exploratory, or short-term studies.

Moreover, there is a lack of direct head-to-head comparative trials examining different intervention families such as cognitive-behavioral, mindfulness-based, and psychophysiological approaches under similar conditions. This gap makes it difficult to determine whether observed differences in outcomes are attributable to intervention type or to contextual and methodological variations across studies.

Importantly, these methodological characteristics do not diminish the conceptual or applied value of stress-regulation research in sport. Rather, they reflect the evolving and context-sensitive nature of applied sport psychology. Nevertheless, greater standardization of intervention protocols, increased use of controlled comparative designs, and integration of multidimensional outcome measures would strengthen the robustness and translational applicability of future research in this field.

## Conclusion

This mini-review highlights the growing role of integrative psychological interventions in supporting stress regulation within competitive sport contexts. Cognitive-behavioral, mindfulness- and acceptance-based, and psychophysiological technology-assisted approaches collectively address the cognitive, emotional, and physiological dimensions of stress, reflecting a multidimensional perspective on performance under pressure.

However, the current evidence base remains heterogeneous, and the effectiveness of these approaches does not appear to be uniform across different sport contexts or athlete populations. Rather than identifying a single optimal intervention, the findings suggest that stress regulation strategies are most effective when they are aligned with the specific demands of the performance environment and the individual characteristics of the athlete.

Importantly, while contemporary technology-assisted approaches offer promising opportunities for real-time monitoring and personalized feedback, their role in psychological skill development requires careful consideration. These tools may enhance awareness and support regulation processes, but they should not replace the development of foundational self-regulation skills. A balanced integration of traditional psychological methods and emerging technologies may therefore represent the most effective approach in applied sport settings.

Overall, stress regulation may be best understood as a trainable and context-sensitive psychological skill that evolves through the interaction of cognitive, emotional, and physiological processes. Future progress in this field will depend on the development of more integrative, individualized, and ecologically valid intervention models, as well as on a deeper understanding of how different approaches interact across diverse sport contexts.
